# Developing a new class of engineered live bacterial therapeutics to treat human diseases

**DOI:** 10.1038/s41467-020-15508-1

**Published:** 2020-04-08

**Authors:** Mark R. Charbonneau, Vincent M. Isabella, Ning Li, Caroline B. Kurtz

**Affiliations:** Synlogic, Inc., 301 Binney Street, Cambridge, MA 02142 USA

**Keywords:** Synthetic biology, Microbiome, Molecular medicine

## Abstract

A complex interplay of metabolic and immunological mechanisms underlies many diseases that represent a substantial unmet medical need. There is an increasing appreciation of the role microbes play in human health and disease, and evidence is accumulating that a new class of live biotherapeutics comprised of engineered microbes could address specific mechanisms of disease. Using the tools of synthetic biology, nonpathogenic bacteria can be designed to sense and respond to environmental signals in order to consume harmful compounds and deliver therapeutic effectors. In this perspective, we describe considerations for the design and development of engineered live biotherapeutics to achieve regulatory and patient acceptance.

## Abstract (web summary)

The role microbes play in human health and the ability of synthetic biology to engineer microbial properties opens up new ways of treating disease. In this perspective, the authors describe the design and development of these living therapeutics.

## Introduction

The human body is host to diverse microbial communities, and the complex interactions between the host and its microbial counterparts play a key role in human health and disease^1^. Notably, members of the microbial community inhabiting the human gastrointestinal tract (termed the gut microbiota) contribute to several metabolic and immune-mediated diseases, including obesity^2^, malnutrition^3^, intestinal inflammatory disease^4^, as well as to anti-cancer immunity^5,6^. The discovery of these host–microbe interactions presents the opportunity to address disease by modulating the structure and function of the gut microbiota. The field of synthetic biology applies the principles of molecular biology and metabolic engineering to design biological circuits that can be applied to medicine. A wide array of tools has been developed for several microbial host organisms, or chassis, that enable investigators to engineer mechanisms to address disease^7^. Engineered bacterial strains can be designed to sense and respond to environmental signals within the body, including those in the gastrointestinal tract or in the microenvironment of solid tumors^8,9^. In this Perspective, we describe the opportunities for and challenges facing the application of synthetic biology tools to the development of therapeutics for human disease. We explore regulatory considerations for the development of engineered live biotherapeutic organisms as medicines and discuss strategies for how these therapeutics can be evaluated for their pharmacokinetic and pharmacodynamic properties. Lastly, we address considerations for manufacturability of engineered microbes to enable production at scale, as well as formulations and presentations that support the needs of patients.

## Regulatory considerations for live biotherapeutic products

Live biotherapeutic products (LBPs) are defined as live organisms designed and developed to treat, cure, or prevent a disease or condition in humans^10^. Notably, LBPs exclude vaccines, filterable viruses, oncolytic viruses, and organisms used as vectors for transferring genes into the host. LBPs are distinguished from probiotic supplements on the basis of their labeling claims, as most probiotics are regulated as dietary supplements and cannot make claims to treat or prevent disease^10–12^. However, some probiotics may fit the definition of LBPs and can be developed as such if they have potential efficacy with respect to disease. LBPs can include genetically modified organisms (recombinant LBPs) if they have been engineered by adding, deleting, or altering genetic material within the organism^10^. In both the United States and Europe, development of LBPs requires the demonstration of quality by establishing safety, reliability, robustness, and consistency of each batch produced^10,13^. They must also be studied in well-controlled clinical trials in the intended patient population to establish safety and efficacy^10–12^.

In the United States, recombinant LBPs are regulated by the Food and Drug Administration (FDA) through the Center for Biologics Evaluation and Research (CBER). While there have been numerous probiotics approved as nutritional supplements and some engineered bacterial strains have been studied in the clinic^14–16^, the FDA has not approved a live biotherapeutic product for medicinal use to date. In 2016, the FDA issued a guidance document describing the regulatory considerations for conducting clinical trials with LBPs^10^. During development of engineered bacterial strains for therapeutic applications, the microorganism must be well characterized and must be evaluated in clinical trials conducted under an investigational new drug application (IND). Regular interaction with regulatory authorities is beneficial, as there is minimal precedent in the field, and the current regulatory guidance documents are very general. For example, the FDA does not provide specific recommendations based on the site of action or therapeutic indication the LBP is intended to treat. Each LBP will have unique properties, including colonization, clearance, microbial products, and delivery modalities (e.g., oral, topical, or injectable). These factors may result in different requirements to demonstrate that the LBP is safe and efficacious^10^. Bacterial components of the chassis, such as lipopolysaccharides, are of less concern for an oral therapeutic but may have significant ramifications for safety when delivered systemically or intratumorally. In some cases, minimal toxicology studies may be needed, if the agent is not disseminated from a local site. However, if there is a risk that the organism may reach other tissues, additional studies could be required to support the safety of the LBP. Since there is no published guidance that outlines toxicology requirements for LBPs specifically, the path for development of a particular clinical candidate must be discussed with the regulatory authorities in the region or country for the intended development and use of the product.

To be approved for medicinal use, the facility in which the microorganism is manufactured, processed, and packaged should operate under regulations of current good manufacturing processes (cGMP). The specific requirements for development of engineered live bacterial therapeutics in the European Union remain to be defined and may differ from those in the United States. The European Pharmacopeia published a monograph setting the quality standards for LBPs for human use, in European Pharmacopoeia, Supplement 9.7; effective in April 2019^13^.

Specific additional considerations concerning the clinical development of engineered bacterial therapeutics include the following: (1) The genetic sequence of exogenously introduced genes, including a high-quality, complete genome sequence for the engineered clinical candidate strain, may be provided to regulators, together with evidence supporting the stability of strain modifications over time. (2) It is highly preferable that the engineered organism be unable to horizontally transfer antibiotic resistance cassettes to other members of the resident microbiota. One way to address this concern is to eliminate all known or suspected antibiotic resistance genes used in the creation of the strain or present in the chassis microorganism. (3) The ability of the organism to replicate or persist in the host and/or the environment can also be characterized, and it may be beneficial to incorporate biocontainment strategies to restrict replication of the candidate strain within the body. (4) Regardless of the inclusion of biocontainment strategies, the residence time and elimination of the engineered organism within the body should be determined. One approach to better characterize the strain is to first study clearance of the orally administered chassis organism in feces of non-human primates and healthy volunteers^17^. (5) Lastly, the biodistribution of the engineered organism outside its target site (e.g., the gastrointestinal tract or solid tumors) may be important to determine.

## Design of engineered therapeutic strains for the human gut

Many bacterial species have been evolutionarily selected for metabolic function within the mammalian gastrointestinal tract, and some probiotic organisms have a long history of safe use in humans^18,19^. Engineered LBPs may be designed to sense and respond to features of the gut environment and represent an opportunity to influence host biology in situ. Engineered bacterial therapeutics can also incorporate biocontainment strategies, such as auxotrophies that limit bacterial replication in the absence of a provided metabolite. More sophisticated approaches for engineered biocontainment have been conceived^20^, but have yet to be deployed in therapeutic applications. In some instances, it is preferable to limit bacterial residence and replication to promote predictable and reproducible pharmacologic properties of the engineered therapeutic. A non-colonizing strain, coupled with a mechanism of biocontainment, may be well suited to achieve this goal.

*Escherichia coli* Nissle 1917 (EcN) has been used as a probiotic since its isolation over 100 years ago^21^. In its unengineered form, EcN has been used to treat various gastrointestinal conditions, including inflammatory bowel disease and irritable bowel syndrome^18,19^. EcN is believed to impede the growth of opportunistic pathogens, including *Salmonella* spp. and other coliform enteropathogens, through the production of microcin proteins or production of iron-scavenging siderophores^18,22,23^. Additionally, EcN may interact with the intestinal epithelium to stimulate anti-inflammatory activities^24^, as well as to restore and maintain intestinal barrier function^25^. Notably, EcN does not exhibit long-term colonization in healthy humans after oral administration^17^. This is likely due to ecological stability of the human gut microbiota and exclusion of incoming new bacteria through a phenomenon termed colonization resistance^26^.

An additional advantage of EcN as a chassis organism for engineered biotherapeutics is the wealth of knowledge about transcriptional and translational control of gene expression in strains of *E. coli*. This knowledge can be leveraged to engineer EcN to respond to the environment within the human gastrointestinal tract. For example, several anaerobic-inducible promoters have been characterized in *E. coli*^27^, which allow for induction of engineered circuits in the anoxic gut environment, without undesired activation during production of biomass. In some instances, it may be preferable to activate effector functions under more specific conditions, rather than constitutively throughout the gastrointestinal tract. For example, coupling gene expression to biosensors of reactive oxygen and nitrogen species for the treatment of inflammatory bowel disease may help deliver effectors specifically where activity is beneficial^7,28^. Regulating effector expression in response to bacterial quorum sensing molecules^29^, pH^30^, specific carbon sources^31–33^, temperature^34^, or combinations of these signals may allow for exquisitely tuned effector functions in various intestinal microenvironments^7^. Mining the extensive transcriptomic data available in *E. coli* can also provide information on endogenous promoters capable of sensing and responding to such signals, which can subsequently be engineered to regulate specific effector functions^7,29,35^.

Several genetically modified EcN strains have been developed as intestinal-acting antimicrobial agents and evaluated in preclinical models. For example, Hwang et. al. demonstrated that the EcN-based gastrointestinal delivery of anti-biofilm enzyme, dispersin B (DspB), resulted in a reduction of pre-colonized *P. aeruginosa* abundance in both nematode and murine models^29^. Other groups have reported the successful production of antimicrobial peptides from EcN that are effective for significantly decreasing murine colonization by Enterococcal species^36^ or *Salmonella typhimurium*^28^. In the face of increasingly prevalent antibiotic-resistant pathogens, novel EcN-based antimicrobials offer promise for the treatment of infections caused by organisms recalcitrant to traditional approaches. In addition, engineered bacterial therapeutics have the potential for increased specificity compared to broad-spectrum antibiotics, as these drugs may be tailored to target particular bacterial genera or species, and their function may be restricted to the gastrointestinal lumen.

Other groups have constructed engineered EcN strains to treat metabolic disorders from within the gut. Chen et al. demonstrated that an N-acylphosphatidylethanolamine (NAPE)-producing strain of EcN could significantly ameliorate symptoms associated with high-fat diet feeding in mice. Mice treated with this recombinant EcN displayed reduced adiposity, insulin resistance, and hepatosteatosis compared to animals treated with an unengineered EcN control^37^. Another group has engineered EcN to express genes responsible for the conversion of fructose, a prevalent sugar in the Western diet that contributes to metabolic disorders and cardiovascular disease, to mannitol, a prebiotic that has been demonstrated to confer protection against metabolic syndrome^38^.

Probiotics in the genera *Lactobacillus* and *Lactococcus* have also attracted significant attention in the engineered biotherapeutic arena. Though the genetic toolbox for these organisms is less advanced than that of *E. coli*, progress has been made with regard to genetic modification and control of gene expression in these organisms^39,40^. Similar to EcN, these genera do not colonize the human gut, thus allowing for predictive pharmacokinetic profiling of therapeutic strains^41,42^. These gram-positive organisms have evolved to survive in the harsh small intestinal environment, and the structure of their cell envelope is advantageous for the secretion of effector proteins into the intestinal milieu. Acto Bio Therapeutics currently has three engineered candidate strains in clinical development using the chassis organism *Lactococcus lactis* (Table 1).

**Table 1:** Engineered bacterial therapeutics currently in clinical development.

Engineered bacterial therapeutic	Chassis organism	Therapeutic indication	Sponsor	Phase of development
AG013	*Lactococcus lactis*	Oral mucositis	Oragenics	Phase 2b
AG014	*Lactococcus lactis*	Gastrointestinal Inflammation in Primary Immunodeficiency	ActoBio Therapeutics	Phase 1
AG019	*Lactococcus lactis*	Type 1 Diabetes Mellitus	ActoBio Therapeutics	Phase 1b/2a
ADXS-HOT	*Lysteria monocytogenes*	Non-Small Cell Lung Cancer	Advaxis Immunotherapies	Phase 1
ADXS-HPV	*Lysteria monocytogenes*	HPV-Associated Cancers	Advaxis Immunotherapies	Phase 1/2
ADXS-PA	*Lysteria monocytogenes*	Metastatic Prostate Cancer	Advaxis Immunotherapies	Phase 2
APS001F	*Bifidobacterium longum*	Solid Tumors	Anaeropharma Science	Phase 1
AZT-04	*Staphylococcus epidermidis*	Cancer Therapy-associated Rashes	Azitra	Phase 1
bacTRL-IL-12	*Bifidobacterium longum*	Solid Tumors	Symvivo	Phase 1
SYNB1020	*E. coli* Nissle 1917	Hyperammonemia	Synlogic	Discontinued
SYNB1618	*E. coli* Nissle 1917	Phenylketonuria (PKU)	Synlogic	Phase 1/2a
SYNB1891	*E. coli* Nissle 1917	Solid Tumors	Synlogic	Phase 1
VXM01	*Salmonella* Typhi Ty21a	Progressive Glioblastoma	VAXIMM	Phase 2

List of engineered bacterial therapeutics in clinical development, describing the chassis organism, therapeutic indication, and the organization sponsoring development.

With recent advances in molecular biology, some groups have turned their attention to bacterial chassis that were not previously amenable to genetic manipulation. For example, CHAIN biotech has developed a modified *Clostridium* strain capable of producing the anti-inflammatory metabolite, β-hydroxybutyrate. This engineered strain can be administered as spores that selectively germinate in the colon to bypass key challenges associated with oral delivery, including survival upon exposure to stomach acids, bile salts, and digestive enzymes. Other groups have focused on organisms that are able to colonize the gastrointestinal tract, including *Bacteroides* spp^43^. This genus of bacteria is known for harboring a diverse repertoire of enzymes for the breakdown of host- and diet-derived carbohydrates^44–47^. Recently, Shepherd et al. engineered *Bacteroides ovatus* to metabolize porphyran, a marine polysaccharide that is rarely encountered in a Western diet^44^. The resulting strain, *B. ovatus* NB001, was shown to stably engraft in the colonic microbiota of mice supplemented with porphyran in the diet, and the fecal abundance of this strain was titratable by modulating dietary porphyran^44^. Novome Biotechnologies is developing this technology for clinical applications, and the use of such strains could be transformative for the treatment of chronic diseases. However, the genetic stability of an engineered live bacterial therapeutic is a concern for organisms that are intended to replicate within and/or colonize the patient’s microbiota, and it may be possible for the strain to transfer engineered genetic material to other members of the endogenous microbiota. Gene cassettes conferring the ability to utilize porphyran, for example, could be horizontally transferred to other members of the gut microbiota. While this genetic transfer is unlikely to be directly harmful to the patient, it may eliminate the competitive advantage of the engineered strain and undermine the efficacy of treatment.

**Fig. 1:**
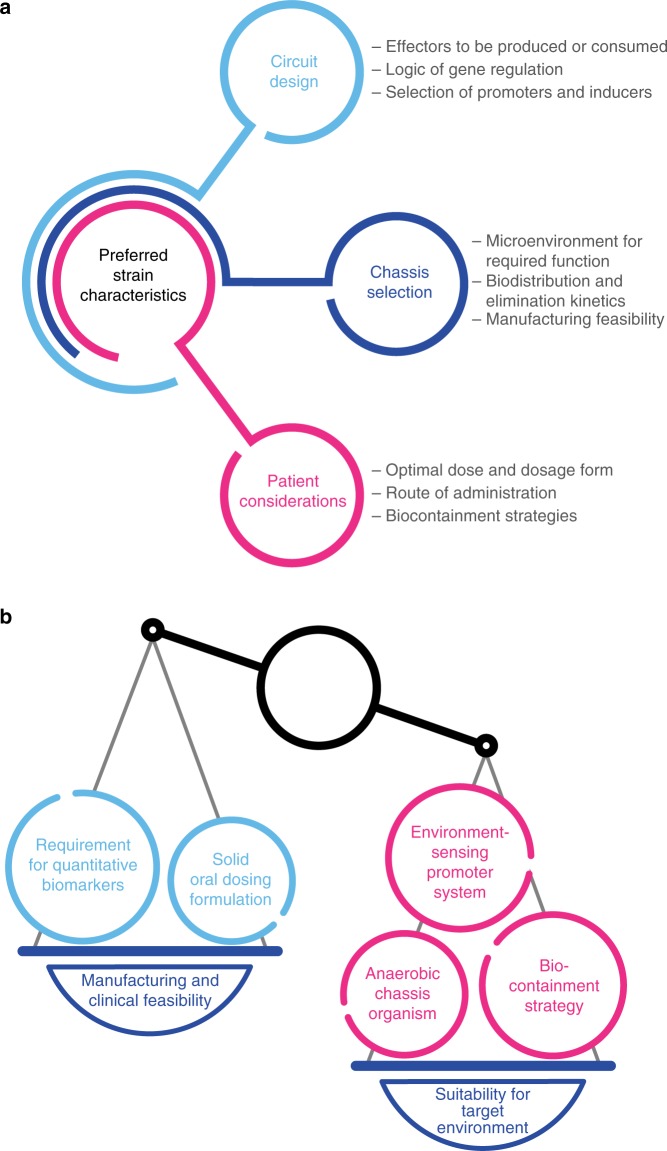
Considerations for the design of engineered live bacterial therapeutics. **a** Several aspects require consideration during the design of an engineered bacterial therapeutic. The selection of a chassis organism can be guided by the desired site of activity and pharmacokinetic properties of the chassis, as well as manufacturing feasibility. The design of genetic circuits may also be influenced by the circuit’s effectors, pragmatic concerns regarding inducer compounds, and the genetic stability of regulatory circuits. Critically, the design of an engineered bacterial drug may also be constrained by considerations for the needs of patients. **b** Optimal strain design often requires a balance between strain suitability for function in the target microenvironment and concerns for feasibility of manufacturing and clinical development.

During selection of a bacterial chassis, as well as during genetic circuit design, the complex biogeography of the gastrointestinal tract can be considered for optimizing activity. Since the human colon is an anaerobic environment^48^, pathways and enzymes that do not require oxygen are preferred. Moreover, while the colon harbors a diverse microbiota, these organisms are predominantly localized to a loosely adherent layer of mucus^49^. Studies of radiolabeled *E. coli* in gnotobiotic mice have demonstrated that mucus-adherent *E. coli* display significantly higher rates of replication than those in the colonic lumen^50^. Interestingly, no differences in replication rates were observed for *Bacteroides thetaiotaomicron*^50^. Strict anaerobes, including *Bacteroides* spp., may be useful chassis organisms due to their abundance in the colonic microbiota of humans, as well as their capacity to consume complex dietary and host-derived glycans^51^. However, the need for strict anaerobiosis may complicate manufacturing of these organisms. The chassis for a bacterial therapeutic thus can be selected to meet both the requirements of its intended function and pragmatic considerations of translation to clinical application (Fig. 1a). These selections in the design and development of engineered LBPs frequently represent trade-offs, as optimization for manufacturing or clinical feasibility may come at a cost to strain function in the body (Fig. 1b). For example, a solid oral dosing formulation may be preferable for an LBP since this format could enable room temperature or refrigerated storage of the product in a patient’s home. However, the process of preparing a lyophilized or spray-dried bacterial powder could result in significant losses to cell viability and/or cell integrity^52,53^. Similarly, incorporation of environmental sensors in the design of an engineered strain may enable exquisite control of engineered gene expression, but these sensors could also severely constrain the acceptable parameters for processes used to prepare biomass. As such, the selection of a chassis organism, the design of engineered gene circuits, and the development of manufacturing processes should be balanced to achieve the required characteristics of an LBP.

Engineered bacterial therapeutics possess a potential advantage over alternative microbiota-directed therapeutic approaches, such as fecal microbiota transplants or defined consortia of naturally occurring species, in that genetic engineering can confer functions that are not expressed by the endogenous microbiota. Engineered LBPs can be designed to perform natural biological processes, such as the assimilation of ammonia into amino acids, at significantly increased rates^14^ and to produce effectors that are not native to bacteria, including human proteins^16^. Functions encoded by engineered bacteria also have potential for the treatment of inborn errors of metabolism (IEMs) present in the host, such as phenylketonuria (PKU)^54^. Patients with PKU harbor genetic mutations that result in reduced activity of the enzyme, phenylalanine hydroxylase, which converts the essential amino acid phenylalanine (Phe) to tyrosine. For PKU patients, dietary protein consumption elevates plasma Phe concentrations, and prolonged elevated plasma Phe can lead to severe cognitive impairment, among other sequelae. Synlogic has engineered a therapeutic strain of EcN, SYNB1618, to degrade Phe by the expression of two distinct mechanisms: (1) the conversion of Phe to *trans*-cinnamic acid by the enzyme phenylalanine ammonia lyase (PAL), and (2) the conversion of Phe to phenylpyruvic acid by the enzyme L-amino acid deaminase (LAAD)^54^. Oral administration of SYNB1618 was shown to significantly lower blood Phe concentrations in a mouse model of PKU, as well as to result in dose-dependent production of the PAL-specific urinary biomarker, hippuric acid, in healthy non-human primates. A recent Phase 1/2a dose escalation study in healthy volunteers and PKU patients that demonstrated that SYNB1618 was generally well tolerated (Clinicaltrials.gov Identifier: NCT03516487). This study also revealed a dose-dependent production of hippuric acid upon administration of SYNB1618, demonstrating Phe consumption by the engineered strain in human subjects.

## Design of engineered therapeutic strains for solid tumors

The notion of treating solid tumors with live bacteria was first reported more than 100 years ago^55–57^. Solid tumors display abnormal blood vessel architecture, resulting in the development of hypoxic regions and a necrotic core that can serve as suitable habitats for obligate and facultative anaerobic bacteria. Preferential colonization of tumors upon administration in mice has been demonstrated for a number of bacterial genera, including *Bifidobacterium*^58^, *Clostridium*^59^, *Salmonella*^60^, and *Escherichia*^9,61^. For example, *E. coli* has been shown to colonize the region surrounding the necrotic core of tumors after intravenous injection^9^, and several reports have demonstrated the use of engineered *E. coli* strains to treat solid tumors in preclinical models^15,62–65^. Zhang et al. developed a strain of EcN to express azurin, a small bacterial protein that induces apoptosis in tumor cells^62^. This engineered strain suppressed the growth of tumors and prevented pulmonary metastasis in mice^63^. Similarly, Li et al. engineered an EcN chassis to produce cytotoxic compounds, including colibactin, glidobactin, and luminmide to suppress tumor growth in a mouse model^64^. More recently, Ho et al. engineered EcN for the treatment of colorectal cancer by expressing HlpA, a protein that binds specifically to a heparan sulphate proteoglycan, enabling engineered EcN to specifically target polyps in a murine colorectal cancer model^65^. The authors combined this polyp-targeting chassis with the secretion of myrosinase, an enzyme that converts glucosinolates, a naturally occurring component of cruciferous vegetables, to the chemopreventive metabolite, sulphoraphane. The combination of HlpA and myrosinase expression led to a significantly enhanced effect on tumor regression and tumor occurrence in mice, compared to a construct expressing myrosinase alone^65^. In another report, Chowdhury et al. engineered a non-pathogenic *E. coli* strain to lyse specifically within the tumor microenvironment and release an anti-CD47 antagonist nanobody. The authors demonstrated the activation of tumor-infiltrating T cells, tumor regression, and long-term survival in a syngeneic tumor model in mice, as well as abscopal effects on untreated tumors^15^.

In addition to strains of *E. coli*, there is precedent for the use of anaerobic organisms for the treatment of cancer preclinically. For example, strains of *Bifidobacterium* have been engineered to express cytosine deaminase (CD) in order to convert the relatively nontoxic compound 5-fluorocytosine (5-FC) into the cytotoxic compound 5-fluorouracil (5-FU) in situ^66^. Co-administration of a CD-expressing *Bifidobacterium infantis* strain with 5-FC significantly inhibited tumor growth in mice^67^. In a similar study, Wei et al. engineered *Bifidobacterium longum* to express the proapoptotic compound, tumstatin^68^. This strain was shown to inhibit tumor growth in a mouse model by various routes of administration^68^. Given the plethora of preclinical data, engineered variants of *E. coli*, *Bifidobacterium*, *Salmonella*, and *Listeria* strains are all currently being evaluated clinically for the treatment of solid tumors (Table 1).

Several aspects are critical for the design of engineered bacterial strains for treatment of tumors, including regulation of engineered circuits, selection of therapeutic effectors, safety and biocontainment within the tumor, and mode of delivery. Notably, these aspects of strain design may interact and have significant implications for the translational potential of engineered live bacterial therapeutics. Chemically inducible promoters, including the tetracycline inducible (Tet) promoter, are widely used in research applications to regulate engineered circuits, due to their ease of use and titratable expression. However, chemically inducible promoters are less amenable to intratumoral applications, since achieving an effective concentration of the inducer molecule in situ may be challenging, and some such compounds are not Generally Regarded As Safe (GRAS) for human use. Another approach to regulation of engineered circuits is through quorum sensing molecules, such as *N*-acyl-homoserine lactones (AHL), that have been studied extensively in *Salmonella* strains^69–73^. In contrast to chemical induction, genetic circuits under the control of oxygen sensitive promoters, such as the fumarate nitrate reductase (FNR)^35^ and the VHb promoter^27^ systems, could obviate the need for exogenously provided inducer compounds. Considering the heterogeneity of tumor architecture, other environmental sensing systems, including temperature-inducible promoters^34^, may provide more consistent induction of engineered circuits. However, temperature-inducible systems possess the significant drawback that circuit expression would not be limited to tumor tissue in the event of systemic strain dissemination.

One advantage of live bacterial therapeutics for treating cancer is that bacterial cells possess inherently proinflammatory properties (e.g., TLR4 stimulation by bacterial lipopolysaccharides^74^). However, the selection of engineered therapeutic effectors is likely to be critical for efficacy in patients. While several examples of effectors have shown promise preclinically, rigorous clinical trials will be required to determine whether these results translate to heterogeneous human cancers that have been recalcitrant to current modes of therapy.

As a new platform for treating cancer patients, the safety of engineered live bacterial therapeutics is paramount, and biocontainment strategies are recommended. An important consideration with respect to safety in patients that may have a compromised immune system is that engineered bacterial products are likely to engage both innate and adaptive immunity in the event of release of the organism into the body following tumor lysis^75^, triggering inflammatory responses. To help address these concerns, nutritional auxotrophies or kill switches can be utilized in engineered strains to prevent replication inside the host organism as well as to control the duration of therapeutic activity and limit the potential toxicity of an engineered strain to patients. A simple “kill switch” strategy is to characterize the antibiotic susceptibility of an engineered live bacterial therapeutic and to use these compounds in the event of suspected bacterial dissemination from tumors.

## Testing strategies for engineered therapeutic organisms

**Fig. 2:**
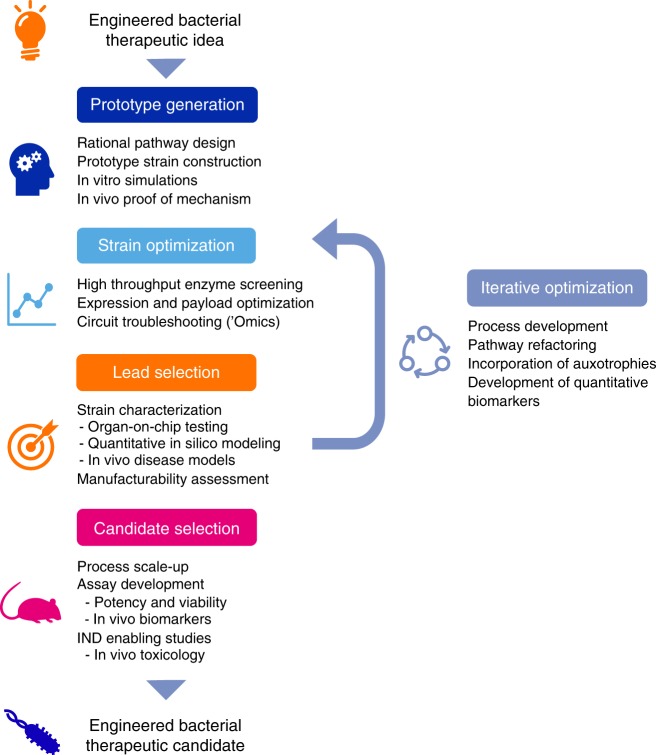
Strategy for the development of engineered live bacterial therapeutic clinical candidates. Schematic representation of a workflow for developing clinical candidate-quality engineered strains. The development workflow should incorporate technologies for optimizing strain potency, as well as predictive in vitro and in vivo assays, as well quantitative pharmacology models, to maximize translational potential for patient populations.

The low cost and high throughput of DNA synthesis and assembly, as well as bioinformatics tools to identify potential targets and effectors, together enable the rapid and cost-effective generation of prototype engineered strains^76^. However, the requirements for clinical development of engineered biotherapeutics, including diligent toxicology studies and adherence to regulatory guidelines, do not scale similarly and represent significant cost and effort for each candidate strain. Therefore, it will be advantageous to develop predictive testing strategies that can be applied in high throughput to characterize the function of engineered strains, to optimize potency, and to establish high confidence in translational potential prior to nomination of strains for clinical development. Figure 2 displays a schematic representation of how this strategy for development of engineered bacterial biotherapeutics could be implemented.

Environmental conditions, including pH, oxygen concentration, and nutrient availability, are major determinants of strain viability and metabolism, and the first line of testing for engineered biotherapeutic organisms could utilize predictive, high throughput in vitro models that recapitulate the physiological conditions of the target environment. For example, methods that simulate the conditions of the human upper gastrointestinal tract, such as the simulated human intestinal microbial ecosystem (SHIME), are useful for characterizing the viability and function of engineered strains^48,77^. These strains can be examined in isolation, or in the context of diverse microbial communities that represent the human gut microbiota. Moreover, these in vitro simulations can elucidate the kinetics of engineered circuit regulation and function over timescales that are relevant to human biology.

A drawback to simplified in vitro simulations is the absence of human cells and tissue architecture. In recent years, significant advances have been made in organ-on-chip microfluidic systems that enable investigators to study the effects of engineered microbes on various human tissues, including the permeability of microbial effectors across epithelial barriers and effects on tissue viability^78^. Recently, Jalili-Firoozinezhad et al. demonstrated the stable co-culture of human intestinal tissues that exhibited an intact mucus layer with a complex human gut microbiota under anaerobic conditions^79^. Though this technology remains in its infancy, and compelling data sets that demonstrate its predictive potential are needed to support its robust application to drug development, organ-on-chip models represent an opportunity to elucidate features of engineered strain function in physiologically relevant environments early in the strain development process.

In vitro models of engineered strain function possess many advantages, including high throughput and comparatively low cost, but they are simplified representations of the host and its associated microbiota. Therefore, animal models will remain a critical component of testing strategies for engineered bacterial therapeutics in the context of various diseases. The selection of an appropriate animal model depends on the question being addressed, as the translational value of animal models varies by species and genotype. For example, while mice are readily available for preclinical studies, the oral bioavailability of small molecule drugs in humans and rodents have demonstrated poor correlation^80^. Human gastrointestinal anatomy and physiology is more closely approximated by pigs and non-human primates than by rodent models^80^, but models of disease states may be unavailable in these large animal species.

Animal models can be applied early in strain development to evaluate performance characteristics of engineered prototypes. For example, rodent models can be used to obtain confidence that a prototype pathway is qualitatively active in vivo. Rodent models are also suitable for determining whether heterologous gene expression results in a substantial fitness defect in an engineered strain compared to the unmodified chassis organism. Such studies can be conducted prior to deploying resources to optimize prototype pathway function (e.g., by screening homologous enzymes or altering gene expression). However, it should be noted that high variability in animal models, together with relatively small study sizes, may impair the statistical power of in vivo studies^81^. As such, quantitative studies of engineered biotherapeutic strain candidates in animals to demonstrate effects on disease states, as well as comparative studies between prototypes, are most appropriately considered only after strain characterization and optimization in vitro. Importantly, these studies should consider the anticipated effect size, as well as variability in the model system, to ensure appropriate design.

The function of engineered biotherapeutic strains in the host environment represents a complex, dynamic system. In the case of oral administration of an engineered organism, strain activity is a function of gastric emptying, changing intestinal pH, oxygen and nutrient availability, strain viability, and dose. Predicting the translational potential of engineered bacterial therapeutics necessitates a move toward mathematical frameworks for integrating data from in vitro and in vivo model systems to predict the behavior of engineered strains in these dynamic conditions. Quantitative systems pharmacology approaches have been widely used to accelerate drug development for other modalities, including small molecules and recombinant proteins, by modeling the pharmacokinetic and pharmacodynamic properties of drug candidates across a wide assortment of therapeutic indications^82^. These approaches will become increasingly important for the design and evaluation of engineered biotherapeutic organisms for clinical development.

## Biomarkers of therapeutic activity

The development of engineered therapeutic strains can be greatly aided by incorporation of robust, quantitative biomarkers of strain function (e.g., metabolites or proteins produced by the engineered strain directly) into strain design. These biomarkers can elucidate the pharmacokinetics and/or pharmacodynamics of the engineered strain and facilitate the translation from preclinical models to clinical studies, enable proof of mechanism during early phase safety studies, and increase confidence in predictions of efficacy for later phase clinical trials. Conversely, the absence of quantitative biomarkers can severely limit the information available to investigators about the function of an engineered live bacterial therapeutic in humans prior to efficacy studies in patients. An ideal biomarker for these purposes satisfies four criteria: (1) the biomarker is mechanistically linked to the designed function of the strain or to the disease itself, (2) the biomarker is quantifiable in noninvasively collected samples (e.g., plasma, urine, or feces), (3) the biomarker has a quantitative relationship to strain activity, and (4) the biomarker compound is readily discernable from endogenous compounds in the sample matrix (i.e., it is unique or produced at levels well above background).

Quantitative biomarkers may not be available for all engineered pathways, however, and the products of some engineered strains may be unstable in host matrices or present at high concentrations endogenously. In some cases, it may be possible to identify metabolic conversions of microbial products that are performed by host tissues. For example, the phenylalanine ammonia lyase (PAL) enzyme expressed by the Phe consuming EcN strain, SYNB1618, produces *trans*-cinnamic acid, which is in turn converted by host tissues into hippuric acid (HA) and excreted in the urine^54^. Measurement of urinary HA provides a quantitative biomarker of strain activity that is directly linked to the strain’s intended function both in preclinical animal models as well as in safety studies with healthy volunteers.

When a biomarker is not readily available from the design of an engineered pathway, in vivo pharmacology studies comparing an engineered biotherapeutic strain to a negative control organism lacking the therapeutic function, together with high throughput data acquisition methods, can be used for putative biomarker identification^83^. These methods could include both targeted and non-targeted metabolomics, proteomics, and high throughput RNA sequencing to identify transcriptional responses. Importantly, any potential biomarker requires rigorous experimental validation to determine whether it satisfies the criteria listed above.

## Manufacturability of engineered live bacterial therapeutics

Due to the unique characteristics of live bacteria, manufacturing engineered bacterial therapeutics differs from other drug modalities in several respects, including development of the manufacturing process, scale-up, and defining critical quality attributes for the drug product. Considerable effort is warranted to develop robust fermentation and downstream processes to balance biomass production and engineered circuit expression. In addition, predictive assays of strain activity are crucial to ensure the potency of engineered bacterial therapeutics. For the purposes of fermentation process development, bench scale bioreactors allow for accurate measurement and control of pH, dissolved oxygen, temperature, as well as the automated addition of nutrients and chemical inducers during cell growth. In recent years, automated parallel bioreactor systems have accelerated fermentation process development by combining smaller volumes and higher throughput to enable greater iteration than traditional benchtop bioreactors^84–86^.

Rational design of engineered bacterial therapeutics should consider compatibility with manufacturing and clinical applications. For example, residual concentrations of chemical inducers may be present after preparation of bacterial drug substance, necessitating additional purification steps if these inducers are used. In addition, not all chemical inducers have received GRAS designation. For this reason, environmental sensors, including oxygen and temperature sensitive regulators, are advantageous.

A key consideration for live bacterial products is cell viability during and after fermentation, downstream processing, formulation, and storage. Traditionally, enumeration of LBPs has relied on agar plating techniques to determine Colony Forming Units (CFU)^10^, and while this methodology remains a staple for the field, it may not be the most appropriate metric for all live bacterial therapeutics. For example, cells in a viable but non-culturable (VBNC) state may be unable to divide and form colonies but may nonetheless retain sufficient metabolic activity to perform some engineered functions in situ^87^. In this case, assessment of viability using commercial live/dead stains to detect intact cell membranes may be more appropriate for enumerating cells in the drug product^88^. By contrast, the expression of multistep metabolic pathways may require actively dividing bacterial cells, making CFU plating more relevant. The ideal approach is most appropriately determined for each engineered clinical candidate strain.

The scale of manufacturing that is necessary for bacterial therapeutics will be determined largely by dosing requirements, and the efficacious dose of an engineered bacterial strain, in turn, is likely to be dependent on both its encoded mechanism of action and its route of administration. For example, metabolic conversions in the gastrointestinal tract may require a larger dose of engineered cells than immunomodulatory mechanisms expressed by intratumorally injected strains. This suggests that there will not be a “one size fits all” solution for the manufacture of engineered live bacterial therapeutics.

An additional aspect that is unique to engineered bacterial drug products is the need to ensure genetic stability during the production of bacterial biomass. The inclusion of engineered genetic circuits encoding novel effector functions may come at a cost to bacterial fitness and/or growth rates, and this can lead to selective pressure for strain variants that have lost the engineered function and its associated fitness costs. To minimize this risk, engineered components can be placed under tight regulatory control to ensure that the engineered gene expression is stably maintained in an “off” state until activation is desired during the preparation of biomass. Robust assays can also be implemented to ensure that engineered circuit function is retained in the drug product.

## Considerations for dosing and formulations

Understanding the needs of the target patient population, as well as practicality in clinical development, can be considered early during the development of an engineered live bacterial therapeutic. Bacterial cells grown in fermenters often must be purified and concentrated to yield a product that is suitable for dosing. For orally administered therapeutics, administration of a frozen suspension may be possible for in-clinic dosing (e.g., in Phase 1 trials). However, for outpatient studies, formulations that require frozen storage and at-home reconstitution present challenges for patients, potentially leading to compliance issues^89^ and risks of product instability. Cold chain storage also presents a challenge for supplying frozen drug products. Therefore, a solid formulation that is stable at room temperature is ideal for an orally administered product. This requires that the live organism can endure processes that convert a liquid culture to a solid form, such as lyophilization or spray drying, to retain viability and potency. Technological advances including microencapsulation and cryoprotectants could improve the stability of future LBP formulations^90,91^, and buffering may be considered to preserve cell activity and viability in the stomach. LBP formulations must also be palatable to patients to ensure compliance with dosing^89^.

For indications that require injection of the engineered live bacterial therapeutic, such as intratumoral administration, these drugs will be reconstituted and administered at a clinic that specializes in this procedure, and frozen liquid formulations are feasible. Hydrogel formulations have also been used for delivery of intratumoral drugs and may improve the concentration of the LBP within the tumor^92^. For dermatological conditions, the LBP may be formulated as a cream or gel so that it can be applied topically by the patient, but engineered bacterial cells will require stability at the storage conditions needed for home use. Odor and color masking may also be needed for any LBP to ensure patient compliance^93^.

A quantitative biomarker of the strain’s activity in the body is also very helpful to bridge early formulations with those used later in development and commercialization. For example, a Phase 1 safety study could be conducted with a frozen liquid preparation of cells to demonstrate activity of the LBP in humans, while a solid oral formulation (e.g., a sachet or capsule) is being developed. Production of the strain-specific biomarker can then be used as a benchmark to bridge to solid formulations before advancing into more lengthy and costly efficacy studies in patients.

## Limitations for developing engineered bacterial therapies

Several challenges and limitations to the development of live engineered bacterial therapeutics are defined not by the tools of synthetic biology but rather by the lack of a clear mechanistic understanding of disease pathophysiology. A quantifiable relationship between the effectors expressed by an engineered organism and the underlying mechanisms of disease is necessary in order to engineer an optimal strain, but there remains a paucity of disease- and effector-related biomarkers to establish dose-response relationships, as well as target engagement at the site of action.

Currently, non-colonizing engineered organisms require frequent dosing, as the residence time of the strain is short in the body^14^. However, it may be desirable to establish longer-term colonization by live bacterial therapeutics in the gastrointestinal tract^44^ to allow for continuous delivery of a therapeutic effector, thereby reducing the need for repeat dosing and enabling lower efficacious bacterial dose levels. This approach could also have the benefit of increasing patient receptivity and compliance, but biocontainment concerns would need to be addressed with regulatory authorities. Understanding the ecological niche occupied by live bacterial therapeutics and how to enhance colonization in the gut is an important area for continued investigation.

Finally, expansion of the tools for synthetic biology, to allow commensal organisms to be considered as chassis for engineered bacterial therapeutics, could enhance compatibility of the strain with the human host. Current bacterial synthetic biology tools are most advanced for strains of *E. coli* and *Lactobacillus*, but there are comparatively few engineering tools for the diverse set of strict anaerobes that reside in the colon.

## Perspective and future developments

Engineered LBPs have the potential for delivering a living medicine that can sense signals within the patient, respond at the site of disease, and alleviate concerns of systemic exposure and toxicity. In other words, engineered microbes could act as biological thermostats and synthesize therapeutic effectors only as needed. To realize this vision, engineered circuits will require validated, disease-specific sensors, tight regulation of engineered genetic circuits, and optimization for maximal potency in order to minimize the required dose of bacterial cells to a patient. The convergence of the tools of molecular biology, low-cost DNA synthesis, and access to various 'omics databases will enable synthetic biologists to build diverse strain libraries, but identifying the most potent constructs will also require high throughput and predictive testing strategies to support the selection of clinical candidates.

As with any new therapeutic modality, patient acceptance of engineered bacterial therapeutics will need to be established. While there has been concern over the use of genetically modified organisms in foods, the use of these organisms in medicines will likely be more acceptable, particularly for treating serious conditions. Indeed, several recombinant bacterial therapeutics are currently being explored clinically (Table 1), and patients are actively enrolling in these trials and accepting the associated risks. Developers of engineered bacterial therapeutics have a responsibility to educate patients, physicians, and the public on their benefits and safety as well as to allay concerns about environmental impacts. As societal awareness of these therapies grows, the public must be assured that the live engineered organisms are contained and cannot disseminate in the environment or undergo further recombination.

Since no recombinant LBPs have been approved for use in humans, the field will be greatly advanced by the first proof of concept demonstration of an engineered bacterial therapeutic delivering a clinically meaningful impact on disease in patients. There will be both successes and failures along the path that will serve as useful lessons to guide the field. Several engineered live bacterial therapeutics are currently entering early or mid-stage clinical development and are poised to deliver the proof of concept needed to unlock the potential for this new class of therapeutics. Once this milestone has been reached, engineered bacterial therapeutics will be rapidly used to combine favorable safety profiles, convenient routes of administration, broad combinations of therapeutic effectors, and scalable manufacturing to create a new category of drugs and, more importantly, to address the significant unmet needs of patients.

## References

[1] Shreiner AB, Kao JY, Young VB (2015). The gut microbiome in health and in disease. Curr. Opin. Gastroenterol..

[2] Turnbaugh PJ (2006). An obesity-associated gut microbiome with increased capacity for energy harvest. Nature.

[3] Blanton LV, Barratt MJ, Charbonneau MR, Ahmed T, Gordon JI (2016). Childhood undernutrition, the gut microbiota, and microbiota-directed therapeutics. Science.

[4] Mazmanian SK, Round JL, Kasper DL (2008). A microbial symbiosis factor prevents intestinal inflammatory disease. Nature.

[5] Matson V (2018). The commensal microbiome is associated with anti-PD-1 efficacy in metastatic melanoma patients. Science.

[6] Gopalakrishnan V (2018). Gut microbiome modulates response to anti-PD-1 immunotherapy in melanoma patients. Science.

[7] Pedrolli DB (2019). Engineering microbial living therapeutics: the synthetic biology toolbox. Trends Biotechnol..

[8] Riglar DT (2017). Engineered bacteria can function in the mammalian gut long-term as live diagnostics of inflammation. Nat. Biotechnol..

[9] Stritzker J (2007). Tumor-specific colonization, tissue distribution, and gene induction by probiotic Escherichia coli Nissle 1917 in live mice. Int. J. Med. Microbiol. IJMM.

[10] U.S. Department of Health and Human Services, Food and Drug Administration Guidance for Industry. Early clinical trials with live biotherapeutic products: Chemistry, manufacturing, and control information. www.fda.gov/downloads/Biologi%E2%80%A6/UCM292704.pdf (2016).

[11] Deal, C. *Science and Regulation of Live Microbiome-Based Products Used to Prevent, Treat, or Cure Diseases in Humans - 09/17/2018 - 09/17/2018*. (U.S. Food and Drug Administration, 2019). http://www.fda.gov/vaccines-blood-biologics/workshops-meetings-conferences-biologics/science-and-regulation-live-microbiome-based-products-used-prevent-treat-or-cure-diseases-humans.

[12] Ross JJ (2008). Considerations in the development of live biotherapeutic products for clinical use. Curr. Issues Mol. Biol..

[13] The European Pharmacopoeia Commission. *Live biotherapeutic products for human use* **9.7** (2019).

[14] Kurtz, C. B. et al. An engineered E. coli Nissle improves hyperammonemia and survival in mice and shows dose-dependent exposure in healthy humans. *Sci. Transl. Med*. **11**, eaau7975 (2019). 10.1126/scitranslmed.aau7975 30651324

[15] Chowdhury S (2019). Programmable bacteria induce durable tumor regression and systemic antitumor immunity. Nat. Med..

[16] Braat H (2006). A phase I trial with transgenic bacteria expressing interleukin-10 in Crohn’s disease. Clin. Gastroenterol. Hepatol..

[17] Kurtz C (2018). Translational development of microbiome-based therapeutics: kinetics of E. coli nissle and engineered strains in humans and nonhuman primates. Clin. Transl. Sci..

[18] Sonnenborn, U. Escherichia coli strain Nissle 1917-from bench to bedside and back: history of a special Escherichia coli strain with probiotic properties. *FEMS Microbiol. Lett*. **363**, fnw212 (2016). 10.1093/femsle/fnw212 27619890

[19] Schultz M (2008). Clinical use of E. coli Nissle 1917 in inflammatory bowel disease. Inflamm. Bowel Dis..

[20] Lee JW, Chan CTY, Slomovic S, Collins JJ (2018). Next-generation biocontainment systems for engineered organisms. Nat. Chem. Biol..

[21] Beimfohr, C. A review of research conducted with probiotic E. coli marketed as symbioflor. *Int. J. Bacteriol.* **2016**, 3535621 (2016). 10.1155/2016/3535621 PMC5138452 27995179

[22] Patzer SI, Baquero MR, Bravo D, Moreno F, Hantke K (2003). The colicin G, H and X determinants encode microcins M and H47, which might utilize the catecholate siderophore receptors FepA, Cir, Fiu and IroN. Microbiology.

[23] Deriu E (2013). Probiotic bacteria reduce Salmonella Typhimurium intestinal colonization by competing for iron. Cell Host Microbe.

[24] Fábrega MJ (2017). Intestinal anti-inflammatory effects of outer membrane vesicles from Escherichia coli Nissle 1917 in DSS-experimental colitis in mice. Front. Microbiol..

[25] Guo, S. et al. Escherichia coli Nissle 1917 protects intestinal barrier function by inhibiting NF-κB-mediated activation of the MLCK-P-MLC signaling pathway. *Mediators Inflamm*. **2019**, 5796491 (2019). 10.1155/2019/5796491 PMC6636522 31354386

[26] Maltby R, Leatham-Jensen MP, Gibson T, Cohen PS, Conway T (2013). Nutritional basis for colonization resistance by human commensal Escherichia coli strains HS and Nissle 1917 against E. coli O157:H7 in the mouse intestine. PLoS ONE.

[27] Khosla C, Bailey JE (1989). Characterization of the oxygen-dependent promoter of the Vitreoscilla hemoglobin gene in Escherichia coli. J. Bacteriol..

[28] Palmer JD (2018). Engineered probiotic for the inhibition of Salmonella via tetrathionate-induced production of Microcin H47. ACS Infect. Dis..

[29] Hwang IY (2017). Engineered probiotic Escherichia coli can eliminate and prevent Pseudomonas aeruginosa gut infection in animal models. Nat. Commun..

[30] Chou C, Aristidou AA, Meng S, Bennett GN, San K (1995). Characterization of a pH-inducible promoter system for high-level expression of recombinant proteins in Escherichia coli. Biotechnol. Bioeng..

[31] Chan PF (2003). Characterization of a Novel Fucose-Regulated Promoter (PfcsK) suitable for gene essentiality and antibacterial mode-of-action studies in Streptococcus pneumoniae. J. Bacteriol..

[32] Letek M (2006). Characterization and use of catabolite-repressed promoters from gluconate genes in corynebacterium glutamicum. J. Bacteriol..

[33] Irani MH, Orosz L, Adhya S (1983). A control element within a structural gene: the gal operon of Escherichia coli. Cell.

[34] Valdez-Cruz NA, Caspeta L, Pérez NO, Ramírez OT, Trujillo-Roldán MA (2010). Production of recombinant proteins in E. coli by the heat inducible expression system based on the phage lambda pL and/or pR promoters. Microb. Cell Factories.

[35] Isabella VM, Clark VL (2011). Deep sequencing-based analysis of the anaerobic stimulon in Neisseria gonorrhoeae. BMC Genomics.

[36] Geldart KG (2018). Engineered E. coli Nissle 1917 for the reduction of vancomycin-resistant Enterococcus in the intestinal tract. Bioeng. Transl. Med..

[37] Chen Z (2014). Incorporation of therapeutically modified bacteria into gut microbiota inhibits obesity. J. Clin. Invest..

[38] Somabhai CA, Raghuvanshi R, Nareshkumar G (2016). Genetically engineered Escherichia coli Nissle 1917 synbiotics reduce metabolic effects induced by chronic consumption of dietary fructose. PLoS ONE.

[39] Guo T, Xin Y, Zhang Y, Gu X, Kong J (2019). A rapid and versatile tool for genomic engineering in Lactococcus lactis. Microb. Cell Factories.

[40] Börner, R. A., Kandasamy, V., Axelsen, A. M., Nielsen, A. T. & Bosma, E. F. Genome editing of lactic acid bacteria: opportunities for food, feed, pharma and biotech. *FEMS Microbiol. Lett*. **366**, fny291 (2019). 10.1093/femsle/fny291 PMC6322438 30561594

[41] Song AA, In LLA, Lim SHE, Rahim RA (2017). A review on Lactococcus lactis: from food to factory. Microb. Cell Factories.

[42] Vesa Pochart, Marteau. (2000). Pharmacokinetics of Lactobacillus plantarum NCIMB 8826, Lactobacillus fermentum KLD, and Lactococcus lactis MG 1363 in the human gastrointestinal tract. Aliment. Pharmacol. Ther..

[43] Mimee M, Tucker AC, Voigt CA, Lu TK (2015). Programming a human commensal bacterium, bacteroides thetaiotaomicron, to sense and respond to stimuli in the murine gut microbiota. Cell Syst..

[44] Shepherd ES, DeLoache WC, Pruss KM, Whitaker WR, Sonnenburg JL (2018). An exclusive metabolic niche enables strain engraftment in the gut microbiota. Nature.

[45] Sonnenburg JL (2005). Glycan foraging in vivo by an intestine-adapted bacterial symbiont. Science.

[46] Xu J (2007). Evolution of symbiotic bacteria in the distal human intestine. PLoS Biol..

[47] Kearney SM, Gibbons SM, Erdman SE, Alm EJ (2018). Orthogonal dietary Niche enables reversible engraftment of a gut bacterial commensal. Cell Rep..

[48] Molly K, Vande Woestyne M, Verstraete W (1993). Development of a 5-step multi-chamber reactor as a simulation of the human intestinal microbial ecosystem. Appl. Microbiol. Biotechnol..

[49] Welch JLM, Hasegawa Y, McNulty NP, Gordon JI, Borisy GG (2017). Spatial organization of a model 15-member human gut microbiota established in gnotobiotic mice. Proc. Natl Acad. Sci. USA.

[50] Li H (2015). The outer mucus layer hosts a distinct intestinal microbial niche. Nat. Commun..

[51] Grondin, J. M., Tamura, K., Déjean, G., Abbott, D. W. & Brumer, H. Polysaccharide Utilization Loci: Fueling Microbial Communities. *J. Bacteriol*. 10.1128/JB.00860-16 (2017). 10.1128/JB.00860-16 PMC5512228 28138099

[52] Bircher L, Geirnaert A, Hammes F, Lacroix C, Schwab C (2018). Effect of cryopreservation and lyophilization on viability and growth of strict anaerobic human gut microbes. Microb. Biotechnol..

[53] Heckly RJ, Quay J (1981). A brief review of lyophilization damage and repair in bacterial preparations. Cryobiology.

[54] Isabella VM (2018). Development of a synthetic live bacterial therapeutic for the human metabolic disease phenylketonuria. Nat. Biotechnol..

[55] Parker RC, Plummer HC, Siebenmann CO, Chapman MG (1947). Effect of histolyticus infection and toxin on transplantable mouse tumors. Proc. Soc. Exp. Biol. Med..

[56] Malmgren RA, Flanigan CC (1955). Localization of the vegetative form of Clostridium tetani in mouse tumors following intravenous spore administration. Cancer Res..

[57] James, N. D. & Sikora, K. Immunotherapy of Tumors. in *Encyclopedia of Immunology* 2nd Edn (ed. Delves, P. J.) 1359–1364 (Elsevier, 1998). 10.1006/rwei.1999.0347.

[58] Fujimori M, Amano J, Taniguchi S (2002). The genus Bifidobacterium for cancer gene therapy. Curr. Opin. Drug Discov. Devel..

[59] Van Mellaert L, Barbé S, Anné J (2006). Clostridium spores as anti-tumour agents. Trends Microbiol..

[60] Lee C, Wu C, Shiau A (2004). Endostatin gene therapy delivered by Salmonella choleraesuis in murine tumor models. J. Gene Med..

[61] Yu YA (2004). Visualization of tumors and metastases in live animals with bacteria and vaccinia virus encoding light-emitting proteins. Nat. Biotechnol..

[62] Yamada T (2004). Apoptosis or growth arrest: modulation of tumor suppressor p53’s specificity by bacterial redox protein azurin. Proc. Natl Acad. Sci. USA.

[63] Zhang Y (2012). Escherichia coli Nissle 1917 targets and restrains mouse B16 Melanoma and 4T1 breast tumors through expression of Azurin protein. Appl. Environ. Microbiol..

[64] Li R (2019). Expressing cytotoxic compounds in Escherichia coli Nissle 1917 for tumor-targeting therapy. Res. Microbiol..

[65] Ho CL (2018). Engineered commensal microbes for diet-mediated colorectal-cancer chemoprevention. Nat. Biomed. Eng..

[66] Nakamura T (2002). Cloned cytosine deaminase gene expression of bifidobacterium longum and application to enzyme/pro-drug therapy of hypoxic solid tumors. Biosci. Biotechnol. Biochem..

[67] Yi C, Huang Y, Guo Z, Wang S (2005). Antitumor effect of cytosine deaminase/5-fluorocytosine suicide gene therapy system mediated by Bifidobacterium infantis on melanoma1. Acta Pharmacol. Sin..

[68] Wei C (2016). Bifidobacteria expressing tumstatin protein for antitumor therapy in tumor-bearing mice. Technol. Cancer Res. Treat..

[69] Din MO (2016). Synchronized cycles of bacterial lysis for in vivo delivery. Nature.

[70] Anderson, J. C., Voigt, C. A. & Arkin, A. P. Environmental signal integration by a modular AND gate. *Mol. Syst. Biol*. **3**, 133 (2007). 10.1038/msb4100173 PMC1964800 17700541

[71] Swofford CA, Van Dessel N, Forbes NS (2015). Quorum-sensing Salmonella selectively trigger protein expression within tumors. Proc. Natl Acad. Sci. USA.

[72] Danino, T., Prindle, A., Hasty, J. & Bhatia, S. Measuring growth and gene expression dynamics of tumor-targeted s. typhimurium bacteria. *J. Vis. Exp.*10.3791/50540. (2013). 10.3791/50540 PMC3731438 23851642

[73] Dai Y, Toley BJ, Swofford CA, Forbes NS (2013). Construction of an inducible cell-communication system that amplifies Salmonella gene expression in tumor tissue. Biotechnol. Bioeng..

[74] Lu Y, Yeh W, Ohashi PS (2008). LPS/TLR4 signal transduction pathway. Cytokine.

[75] Howard SC, Jones DP, Pui C-H (2011). The tumor lysis syndrome. N. Engl. J. Med..

[76] Hughes, R. A. & Ellington, A. D. Synthetic DNA synthesis and assembly: putting the synthetic in synthetic biology. *Cold Spring Harb. Perspect. Biol*. **9**, a023812 (2017). 10.1101/cshperspect.a023812 PMC5204324 28049645

[77] Minekus M (2014). A standardised static in vitro digestion method suitable for food—an international consensus. Food Funct..

[78] Kasendra M (2018). Development of a primary human small intestine-on-a-Chip using biopsy-derived organoids. Sci. Rep..

[79] Jalili-Firoozinezhad S (2019). A complex human gut microbiome cultured in an anaerobic intestine-on-a-chip. Nat. Biomed. Eng..

[80] Hatton GB, Yadav V, Basit AW, Merchant HA (2015). Animal farm: considerations in animal gastrointestinal physiology and relevance to drug delivery in humans. J. Pharm. Sci..

[81] Festing MFW, Altman DG (2002). Guidelines for the design and statistical analysis of experiments using laboratory animals. ILAR J..

[82] Milligan PA (2013). Model-based drug development: a rational approach to efficiently accelerate drug development. Clin. Pharmacol. Ther..

[83] Wishart DS (2016). Emerging applications of metabolomics in drug discovery and precision medicine. Nat. Rev. Drug Discov..

[84] Xu P (2017). Characterization of TAP Ambr 250 disposable bioreactors, as a reliable scale-down model for biologics process development. Biotechnol. Prog..

[85] Sandner V, Pybus LP, McCreath G, Glassey J (2019). Scale-down model development in ambr systems: an industrial perspective. Biotechnol. J..

[86] Manahan, M. et al. Scale-down model qualification of ambr® 250 high-throughput mini-bioreactor system for two commercial-scale mAb processes. *Biotechnol. Prog*. 10.1002/btpr.2870 (2019). 10.1002/btpr.2870 31207168

[87] Zhang, S. et al. Induction of Escherichia coli Into a VBNC state by continuous-flow UVC and subsequent changes in metabolic activity at the single-cell level. *Front. Microbiol*. **9**, 2243 (2018). 10.3389/fmicb.2018.02243 PMC6167417 30319570

[88] Boulos L, Prévost M, Barbeau B, Coallier J, Desjardins R (1999). LIVE/DEAD® BacLight^TM^: application of a new rapid staining method for direct enumeration of viable and total bacteria in drinking water. J. Microbiol. Methods.

[89] Shahiwala A (2011). Formulation approaches in enhancement of patient compliance to oral drug therapy. Expert Opin. Drug Deliv..

[90] Yus, C. et al. Targeted release of probiotics from enteric microparticulated formulations. *Polymers* **11**, 1668 (2019). 10.3390/polym11101668 PMC6835770 31614915

[91] Guowei S (2019). Comprehensive optimization of composite cryoprotectant for Saccharomyces boulardii during freeze-drying and evaluation of its storage stability. Prep. Biochem. Biotechnol..

[92] Fakhari A, Anand Subramony J (2015). Engineered in-situ depot-forming hydrogels for intratumoral drug delivery. J. Control. Release.

[93] Tan X, Feldman SR, Chang J, Balkrishnan R (2012). Topical drug delivery systems in dermatology: a review of patient adherence issues. Expert Opin. Drug Deliv..

